# *In vivo* delivery of VEGF RNA and protein to increase osteogenesis and intraosseous angiogenesis

**DOI:** 10.1038/s41598-019-53249-4

**Published:** 2019-11-28

**Authors:** Robin M. H. Rumney, Stuart A. Lanham, Janos M. Kanczler, Alexander P. Kao, Lalitha Thiagarajan, James E. Dixon, Gianluca Tozzi, Richard O. C. Oreffo

**Affiliations:** 1 Bone and Joint Research Group, Centre for Human Development, Stem Cells and Regeneration, Faculty of Medicine, Southampton University, Southampton, SO16 6YD United Kingdom; 2 School of Pharmacy and Biomedical Sciences, University of Portsmouth, St Michael’s Building, White Swan Road, Portsmouth, PO1 2DT United Kingdom; 3 Zeiss Global Centre, School of Mechanical and Design Engineering, University of Portsmouth, Portsmouth, PO1 3DJ United Kingdom; 4 Wolfson Centre for Stem Cells, Tissue Engineering and Modelling (STEM), Centre of Biomolecular Sciences, School of Pharmacy, University of Nottingham, Nottingham, NG7 2RD United Kingdom

**Keywords:** Protein delivery, Cardiovascular models, Musculoskeletal models, Gene delivery

## Abstract

Deficient bone vasculature is a key component in pathological conditions ranging from developmental skeletal abnormalities to impaired bone repair. Vascularisation is dependent upon vascular endothelial growth factor (VEGF), which drives both angiogenesis and osteogenesis. The aim of this study was to examine the efficacy of blood vessel and bone formation following transfection with *VEGF* RNA or delivery of recombinant human VEGF_165_ protein (rhVEGF_165_) across *in vitro* and *in vivo* model systems. To quantify blood vessels within bone, an innovative approach was developed using high-resolution X-ray computed tomography (XCT) to generate quantifiable three-dimensional reconstructions. Application of rhVEGF_165_ enhanced osteogenesis, as evidenced by increased human osteoblast-like MG-63 cell proliferation *in vitro* and calvarial bone thickness following *in vivo* administration. In contrast, transfection with *VEGF* RNA triggered angiogenic effects by promoting VEGF protein secretion from MG-63_*VEGF*165_ cells *in vitro*, which resulted in significantly increased angiogenesis in the chorioallantoic (CAM) assay *in ovo*. Furthermore, direct transfection of bone with *VEGF* RNA *in vivo* increased intraosseous vascular branching. This study demonstrates the importance of continuous supply as opposed to a single high dose of VEGF on angiogenesis and osteogenesis and, illustrates the potential of XCT in delineating in 3D, blood vessel connectivity in bone.

## Introduction

The temporal and spatial coordination of vascularisation within bone is pivotal for skeletal development including bone formation, maintenance and repair^1^. Endochondral ossification is contingent on the arrival of blood capillaries from the periosteal bud, while, intramembranous ossification is associated with both intussusceptive and sprouting forms of angiogenesis^2^. In bone, the process of angiogenesis is coupled to osteogenesis as new blood vessels form in the wake of bone resorption by osteoclasts and the subsequent chemoattractant recruitment of osteoblast precursors to enable endochondral bone formation^3–6^. Vascular endothelial growth factor (VEGF) was first shown over two decades ago to be the key factor coupling osteogenesis and angiogenesis as inactivation of VEGF concomitantly subdued blood vessel invasion and bone formation^7,8^. VEGF receptors including VEGFR1 (Flt1), VEGFR2 (Kdr), and VEGFR3 (Flt-4) are expressed by both blood vessel forming endothelial cells^9^ and bone forming osteoblasts^10,11^. In vascular endothelial cells, VEGFR2 is the main signalling VEGF receptor^9^, while the co-receptors neuropilin-1 and 2 influence endothelial cell survival, adhesion, and migration^12,13^. Blood vessel formation is dependent upon a complex signalling axis that includes hypoxia inducible factor (HIF)-1 (consisting of HIF-1α and HIF-1β subunits), which drives expression of VEGF that in turn acts upon VEGF receptors^14^. In osteoblasts, VEGFR1-3, neuropillin-1 and -2 are required for stimulation of osteoblast chemotaxis^15^, regulation of osteoblast development^10,11^, and of bone remodelling^10^. In addition to responding to VEGF, osteoblasts are known to be a predominant source of VEGF in bone and release VEGF in response to a range of factors including hypoxia and mechanical strain^1,8^.

Given the central role of skeletal blood vessels in bone formation and maintenance, it is unsurprising that a lack of bone vascularisation has been identified as a contributory factor in a number of bone pathologies. Avascular necrosis of the femoral head has several causes including steroid treatments or alcohol abuse, which reduce the availability of endothelial progenitor cells and result in decreased angiogenesis within bone^16^. Diabetes patients suffer from impaired bone formation and fracture healing^17^, which may be a consequence of diabetes, related cardiovascular disease, which restricts blood supply to the bone^18^. Angiogenesis is pivotal in bone repair where one of the first responses to blunt trauma requires the formation of a hematoma around the fracture site accompanied by a triggering of new blood vessel formation^19^. A failure of angiogenesis around the injury site can result in bone non-union, a serious clinical complication, present in up to 9% of fracture cases^20^. Current treatments are limited and often require invasive surgery, with long recovery times and can be dependent upon the available vasculature within bone grafts^21–23^. As these pathologies and morbidities are associated with a lack of an adequate functional bone vasculature, a suitable approach for treatment necessitates methods of controlling and enhancing angiogenesis in bone.

There are several isoforms of VEGF-A of which VEGF_164/165_ is the most widely studied in bone given the established requirement of VEGF_164/165_ for angiogenesis and bone formation. Maes and co-workers showed mice expressing VEGF_120_ alone, have impaired bone growth with reduced bone vascularisation and decreased expression of osteoblastic genes^24^. In contrast, mice expressing only VEGF_188_ exhibit dwarfism associated with a lack of vascularisation around the epiphysis and impaired cartilage development. Interestingly, the skeletons of mice expressing only VEGF_164_ exhibit normal bone structure and mineralisation demonstrating the importance of this isoform^25^. A raft of studies have confirmed osteoblast specific VEGF expression is particularly important in bone formation and repair. Deletion in osteoblasts of the von Hippel–Lindau gene (*Vhl*), which increases HIF-1α and subsequent VEGF expression, has been associated with increased angiogenesis and volume of long bones^26^. In craniofacial bones, deletion of *Vegfa* reduces osteoblast commitment^27^, while, VEGF derived from osteoprogenitor cells is required for intramembranous ossification independently of vasculogenesis^28^. In an osteoblast specific *Vegfa* knockout mouse model, the absence of osteoblast-derived VEGF was linked to decreased intramembranous bone formation, decreased angiogenesis and a reduction in callus remodelling as assessed using a tibial bone drill defect model^29^.

Given the importance of localised VEGF release in directing the formation of new bone and blood vessels, the current study set out to enhance the innate capacity of bone cell populations to generate VEGF and to quantify the changes in osteogenesis and angiogenesis within bone compared with administration of recombinant human VEGF_165_ protein (rhVEGF_165_). The effects of rhVEGF_165_ were compared with that of *VEGF*_165_ RNA transfection upon angiogenesis *in ovo* and in both bone formation and intraosseous vascularisation *in vivo* analyzed by XCT.

## Results

### Contrasting effects of *VEGF*_165_ RNA transfection and rhVEGF_165_ protein *in vitro*

**Figure 1:**
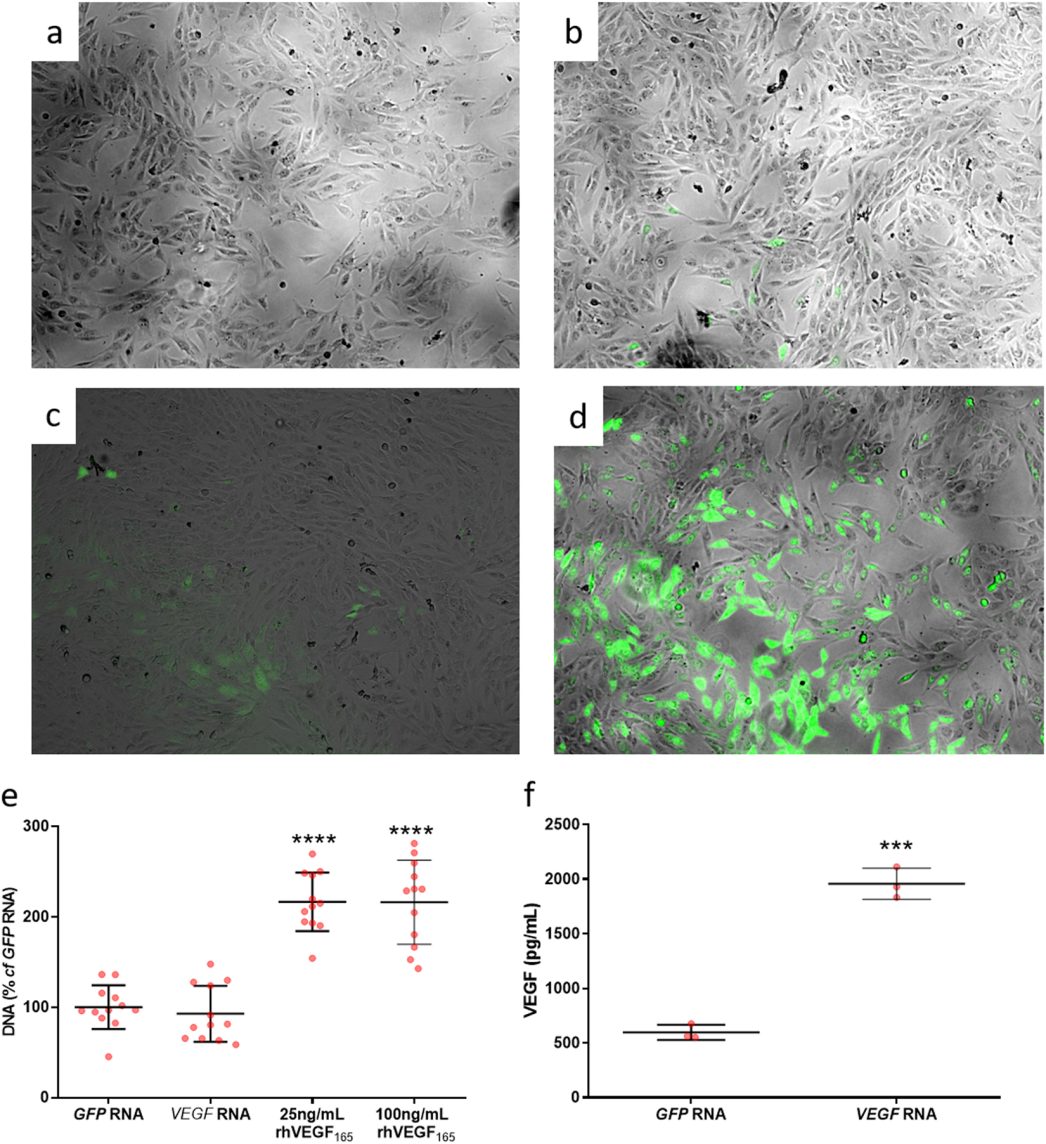
Contrasting effects of *rhVEGF*_*165*_ RNA transfection and rhVEGF_165_ protein *in vitro*. MG-63 cells cultured in 24 well plates were transfected with either *GFP* or *VEGF*_*165*_ RNA, or treated with 25 ng/µl rhVEGF_165_. GFP fluorescence was visualised after 16–20 hours as a positive control for transfection (**a**–**d**). A titration of *GFP* RNA was used from 0.1 µg (**a**), 0.5 µg (**b**), 1 µg (**c**) to 5 µg per well (**d**). Double stranded DNA quantified using the PicoGreen assay was used as a marker for cell proliferation and demonstrated a 116% increase in response to rhVEGF_165_ treatment at both 25 ng/mL and 100 ng/mL (N = 12 in total with 4 wells per treatment group and 3 independent replica plates) (b). VEGF quantified from conditioned media by ELISA was on average 596 pg/mL from MG-63 _*GFP*_ cells and 1958 pg/mL from MG-63 _VEGF1_65 cells (**c**) (N = 3 per treatment group). Statistical analyses were carried out in IBM SPSS Statistics 25 with ANOVA and Tukey post-hoc tests or t-tests where comparing just two treatment groups (***P < 0.001, ****P < 0.0001).

Transfection with *GFP* RNA engendered the production of GFP protein, visible under fluorescent microscopy in a dose dependent manner (Fig. 1a–d). Transfection with 0.1 and 0.5 µg per well was insufficient to generate detectable GFP expression (Fig. 1a,b, respectively). Transfection with 1 µg and 5 µg per well engendered GFP that was visible under fluorescent microscopy (Fig. 1c,d, respectively). Treatment of human osteoblast-like MG-63 cells with 25 ng/mL or 100 ng/mL rhVEGF_165_ protein for 24 hours resulted in a significant increase (116.4% and 116.0% respectively; P < 0.0001) in DNA content compared to non-treated controls as measured using the PicoGreen assay. In contrast, transfection with *VEGF*_165_ RNA had no significant effect on proliferation (Fig. 1e). Consistent with these observations, transfection of MG-63 cells with 1 µg per well of *VEGF*_165_ RNA significantly increased secretion of VEGF protein by 228% (MG-63_*GFP*_ = 596 ± 69.5 pg/mL, MG-63_*VEGF165*_ = 1958 ± 142.2 pg/mL, P < 0.001 Fig. 1f).

### Effect of *VEGF*_165_ RNA transfected MG-63 cells upon angiogenesis in the CAM assay

**Figure 2:**
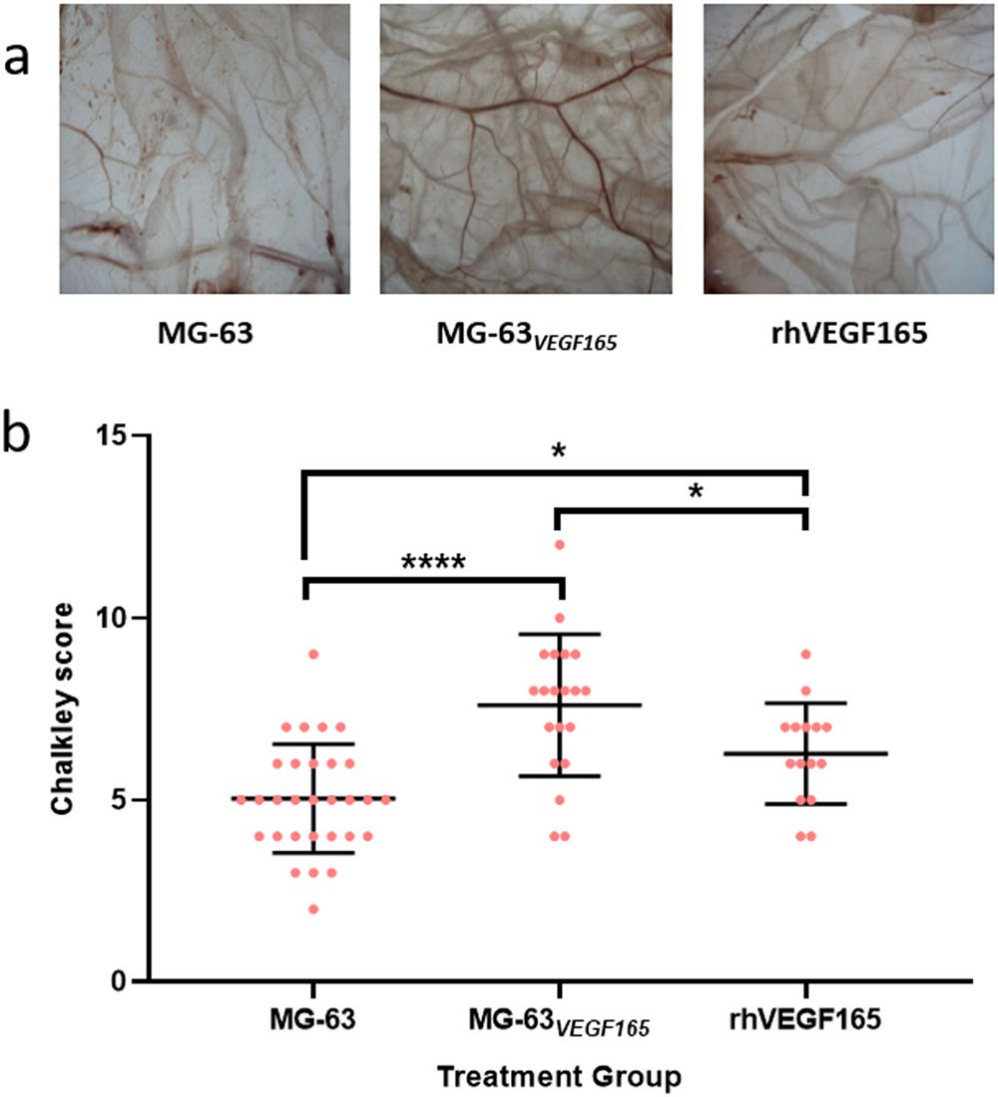
Vessel density is increased by transfected MG-63 cells and rhVEGF_165_ in the CAM assay. CAM assays were initiated on chicken eggs 10 days after fertilisation. Electrospun PCL scaffolds seeded with MG-63_*GFP*_ or MG-63_*VEGF165*_ cells were placed on the CAM and additional eggs were treated with rhVEGF_165_ (10 µL of 1 µg/mL solution). After 7 days, the CAM membranes were harvested for quantification of angiogenesis with a dissecting microscope under which differences in treatment group were clearly visible (A). Angiogenesis was quantified from 5 regions per CAM using a Chalkley graticule in the eyepiece of the dissecting microscope. Mean Chalkley scores for each treatment group were 5.0 for membranes with MG-63 seeded PCL scaffolds (N = 6), 7.6 with MG-63_*VEGF165*_ seeded PCL scaffolds (N = 4), and 6.3 with rhVEGF_165_ treatment (N = 3). Statistical tests were carried out in IBM SPSS statistics 25 using univariate analysis and Tukey post-hoc tests (*P < 0.05, ***P < 0.001).

Following *in vitro* demonstration of modulation of VEGF production in human osteoblast-like MG-63 cells, angiogenesis was examined using *VEGF*_165_ RNA transfected MG-63 cells in the CAM assay. Angiogenesis was quantified from five regions within individual CAM membranes using the Chalkley assay (representative images in Fig. 2a). The Chalkley score for angiogenesis in CAM assays following culture with polycaprolactone (PCL) scaffolds seeded with MG-63 cells was 5.0 ± 1.50. In contrast, transfected

MG-63_*VEGF*165_ cells on PCL scaffolds placed on the CAM demonstrated significantly enhanced angiogenesis (Chalkley score 7.6 ± 1.96; P < 0.001). The addition of a single dose of 10 µL of rhVEGF_165_ (1 µg/mL) resulted in enhanced angiogenesis (Chalkley score 6.3 ± 1.39; P < 0.05, Fig. 2b) although this did not reach the same level as the MG-63_*VEGF165*_ group.

### Direct delivery of rhVEGF_165_ protein increases bone volume

**Figure 3:**
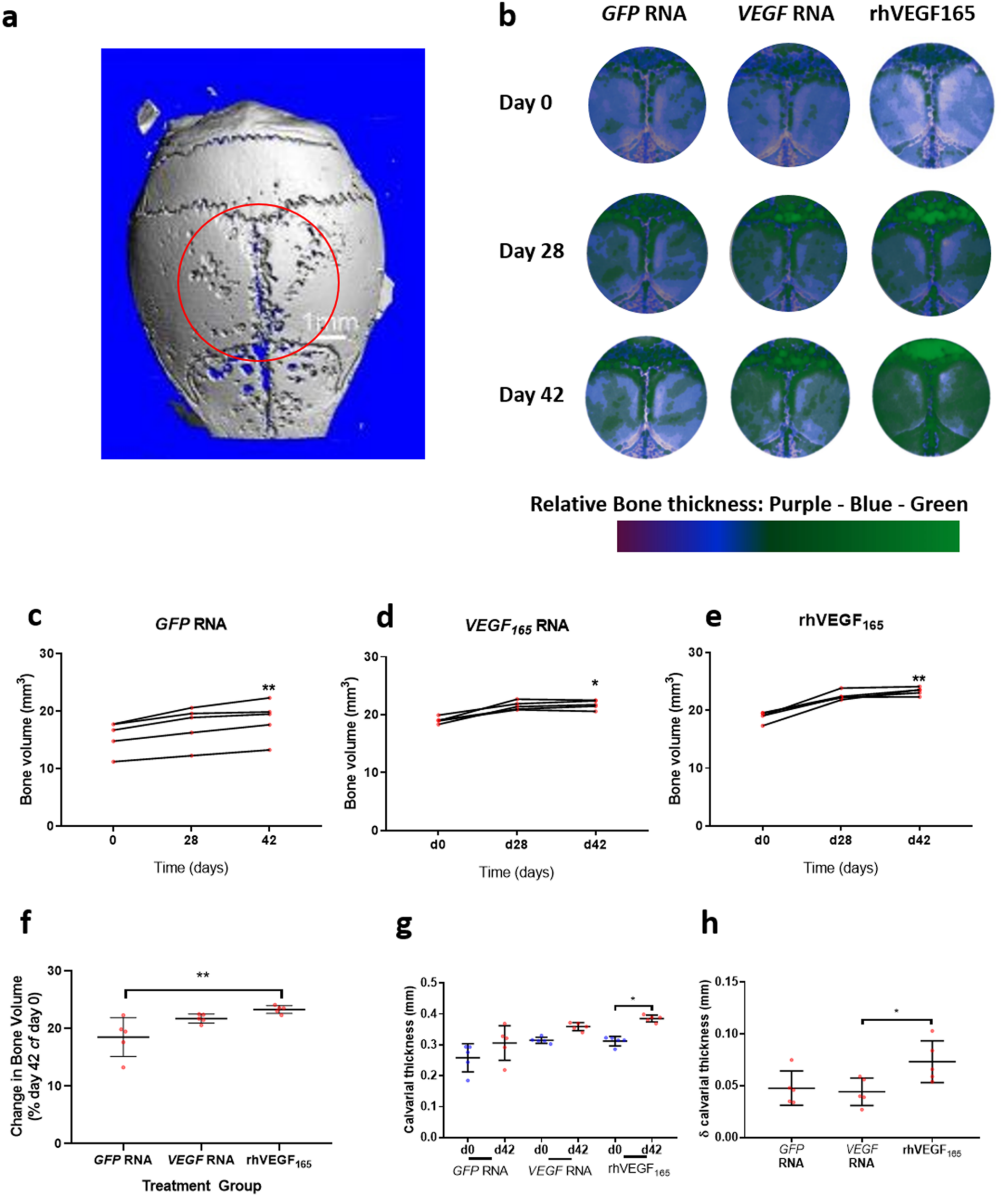
Bone volume is increased following direct *in vivo* treatment with rhVEGF_165_. Murine calvariae were directly transfected with *GFP* or *VEGF*_*165*_ RNA or treated with rhVEGF_165_ protein *in vivo* in the region indicated (**a**). Calvariae were scanned by *in vivo* µCT on days 0, 28 and 42. Colour coded reconstructions show thinner bone in purple and thicker bone in green between treatment groups and across the time course (**b**). Changes in bone volume were recorded in the ROI in all treatment groups along the 42-day time course (**c**–**e**). rhVEGF_165_ protein treated calvariae demonstrated the highest percentage increase in bone volume (**f**), significantly increased bone thickness (**g**) and the greatest increase in thickness (**h**) (N = 4–5 mice per treatment group). Statistical tests were carried out in GraphPad Prism with ANOVA and Dunn’s multiple comparison or Tukey post-hoc tests (*P < 0.05, **P < 0.01).

Following evidence of enhanced angiogenesis *in ovo*, angiogenesis was examined using an *in vivo* murine calvarial model. Mice with calvariae treated with rhVEGF_165_ protein or transfected with *GFP* or *VEGF*_165_ RNA *in vivo* were scanned by µCT on the day of treatment, after 28 days and, after 42 days whereupon the experiments were stopped. Changes in bone formation (region of interest) were examined across the central area of the calvariae over the parietal bones and sagittal suture corresponding to the site of transfection (Fig. 3a). Changes in bone thickness were recorded over time and visualised using colour coded reconstructed images with relatively thinner bone identified in purple and, dense bone in green (Fig. 3b). Analysis by *in vivo* µCT revealed significant increases in calvarial bone volume in mice treated with *GFP* RNA (Fig. 3c, P < 0.01), *VEGF*_165_ RNA (Fig. 3d, P < 0.05) and rhVEGF_165_ protein (Fig. 3e, P < 0.01). Changes in bone volume varied across groups in the time frame of the study. *GFP* RNA transfected calvariae increased in volume by 18.51%, while mice transfected with *VEGF*_165_ RNA increased by 21.75% and mice treated with rhVEGF_165_ displayed a significant increase in bone volume of 23.31% (P < 0.05 cf *GFP* RNA transfected controls, Fig. 3f).

Calvarial bone thickness increased from an average of 0.3122 ± 0.0151 mm on day 0 to 0.3845 ± 0.0112 mm on day 42 in rhVEGF_165_ treated animals (P < 0.05). There were no significant increases in calvarial bone thickness between 0 and 42 days in either the GFP RNA or the *VEGF*_165_ RNA treated mice (Fig. 3g). The mean increase in bone thickness between 0 and 42 days for rhVEGF_165_ treated animals was 0.0732 ± 0.0202 mm, significantly more than the value of 0.0442 ± 0.0132 mm observed in *VEGF*_165_ RNA treated mice (Fig. 3h).

### Transfection with *VEGF*_165_ RNA increases intraosseous vascular branching *in vivo*

**Figure 4:**
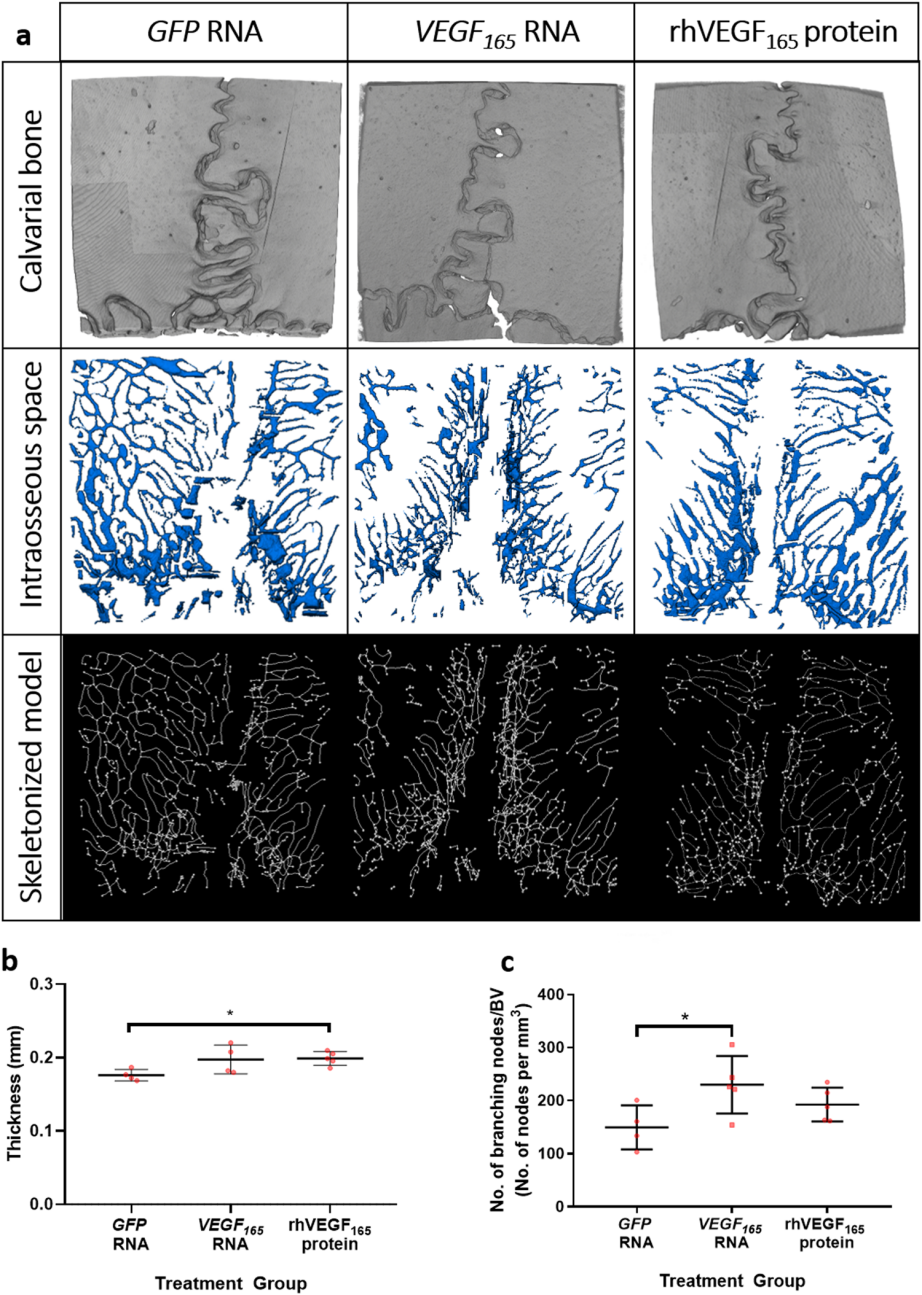
Intraosseous vascular branching is increased by *in vivo* transfection with *VEGF*_*165*_ RNA. Isolated murine calvariae were scanned *ex vivo* by XCT and each ROI was analyzed in Avizo 9.3 to generate detailed 3D reconstructions of the calvariae, the intraosseous space and skeletonized models of the vascular connectivity (**a**). Calvarial thickness was quantified by XCT (**b**). The number of branching nodes was normalised to bone volume to reveal increased intraosseous vascular branching following *in vivo* transfection with *VEGF*_*165*_ RNA (**c**). Statistical tests were carried out in GraphPad Prism using ANOVA and Dunnett’s post-tests (*=< P0.05).

Reconstructed images from high resolution XCT allowed visualisation of bone topography, intraosseous vascular space and vascular branching (Fig. 4a). Calvarial thickness, bone volume and intraosseous space were mapped in 3-dimensions using Avizo software and the number of branching nodes quantified from a skeletonized map of the intraosseous vascular cavities.

Calvarial thickness recorded from each region of interest (ROI) by XCT was 0.1760 ± 0.0079 mm from *GFP* RNA transfected mice and 0.2095 ± 0.0092 mm from rhVEGF_165_ treated mice (P < 0.05 *cf GFP* RNA transfection). Calvarial thickness measured by XCT from *VEGF* RNA transfected mice was 0.1975 ± 0.0197 mm (Fig. 4b).

The mean density of branching nodes, normalised to bone volume in each ROI, was 149.6 ± 41.5 per mm^3^ in calvariae from *GFP* RNA transfected mice and observed to be significantly increased in VEGF_165_ RNA transfected mice (230.1 ± 54.1 per mm^3^; P < 0.05 cf *GFP* RNA transfection). In contrast, within the calvariae of rhVEGF_165_ treated mice the mean value was 192.6 ± 31.9 per mm^3^ and was not significantly different to the other treatment groups (Fig. 4c).

## Discussion

The central aim of the present study was to examine the different effects of rhVEGF_165_ protein and transfected *VEGF*_165_ RNA upon angiogenesis and bone formation. The current study shows that rhVEGF_165_ protein produced, overall, an osteogenic effect with increased proliferation of human osteoblast-like MG-63 cells at 25 ng/mL and 100 ng/mL *in vitro* (Fig. 1e) corresponding to a previous study where matching concentrations of rhVEGF_165_ decreased trabecular spacing, increased bone volume and trabecular number in organotypic cultures embryonic chick femurs^30^. Consistent with these *in vitro* findings, treatment with rhVEGF_165_ enhanced calvarial bone thickness *in vivo* compared to other treatment groups (Figs. 3g,h and 4b). Differences in osteogenesis within calvariae were quantifiable within a longitudinal *in vivo* µCT study, which allowed direct comparison between bone thickness and volume within individual mice at specific time points to counteract any variance in bone parameters within treatment groups (Fig. 3). Calvariae were further analyzed *ex vivo* by XCT to generate higher resolution scans over a smaller ROI compared to *in vivo* µCT. This technique demonstrated that calvariae from rhVEGF_165_ treated mice were significantly thicker than *GFP* RNA controls (Fig. 4b)

Although dosages of between 12.5 and 50 ng/mL of rhVEGF_165_ have previously been shown to increase tubule formation from epithelial cells *in vitro*^31^, it was transfection with *VEGF*_165_ RNA which promoted, predominantly, angiogenesis with increased VEGF protein secretion *in vitro* (Fig. 1f) and an increase of angiogenesis in the CAM assay that was stronger than observed with rhVEGF_165_ protein (Fig. 2a,b). Direct transfection of *VEGF*_165_ RNA into calvarial bones *in vivo* was found to increase branching of the intraosseous vascular cavity, quantified via a novel approach using X-ray computed tomography (Fig. 4c).

The contrasting effects of rhVEGF_165_ protein and transfection with *VEGF*_165_ RNA can be better understood in the context of prior investigations into VEGF delivery. Previous studies have indicated that the precise delivery mechanism employed can alter the effects of VEGF on bone *in vivo* with some approaches increasing either osteogenesis or angiogenesis with other approaches promoting both. Subcutaneous injection of rhVEGF_165_ within a matrigel scaffold to an *in vivo* mouse model enhanced angiogenesis concomitantly with new bone formation^32^, partially consistent with the present data on calvarial bone formation but not angiogenesis following *in vivo* delivery of rhVEGF_165_. VEGF carrying adenovirus has been used for direct *in vivo* localised injection to increases osteoblast number and osteoid volume^33^. In contrast, the *in vivo* delivery of *VEGF*_165_ plasmid freeze dried into deproteinized bone and implanted into rabbit femoral drill defect, only increased formation of new capillary vessels with no reported changes in bone volume^34^. This latter study parallels our own findings, whereby transfection increased intraosseous angiogenesis (Fig. 4c). Interestingly, rhVEGF_165_ increased bone thickness *in vivo* without significantly increasing intraosseous angiogenesis, while transfection with *VEGF*_165_ *RNA* produced what appeared to be almost the reverse effect. Possible explanations may include differences in the relative abundance of VEGF receptors on bone cell populations required for angiogenesis versus osteogenesis. Interestingly, the expression pattern of VEGF receptors in response to VEGF protein has previously been shown to be higher in co-cultures of human umbilical vein endothelial cells and human foetal diaphyseal/epiphyseal foetal femur cells compared to expression following individual cell culture^30^. It cannot be excluded that the dose response to VEGF varies according to cell type, with a continuous low-level release of VEGF following transfection potentially more advantageous to angiogenic cells while a large single dose of VEGF protein could trigger osteoblast activity. Furthermore, it is possible that different cell populations may be more resilient to transfection than others with the resultant VEGF effects that would accompany. The ease of access of protein and RNA may be confounding variables given differences in size, charge, shape and stability all of which could affect subsequent permeation and angiogenic or bone cell population interactions and responses. Resolving these differences will ultimately inform treatment strategies and this area requires further investigation.

The dosages of RNA and protein that engendered increases in vascularisation during the CAM assay were applied to murine calvariae *in vivo*, despite this, only *VEGF* RNA increased angiogenesis in the calvarial assay. It is of course plausible that a particular concentration of rhVEGF_165_ protein that has a pro-angiogenic effect in the CAM may not have the same effect in an *in vivo* calvarial model as our data show. An alternative could be to carry out dose responses *in vivo* and future studies could test additional concentrations. However, for the purposes of this study, and to try and address and meet the principles of the 3Rs (Replacement, Reduction and Refinement) it was decided not to carry out full dose responses *in vivo* which would have multiplied the numbers of animals required.

Quantification of vasculature within bone is a substantial challenge. Histological sections are typically too thin to enable the visualisation in three dimensions of the patterning of the bone vasculature. Generation of thicker sections and immunofluorescent labelling has enabled visualisation of bone vasculature in improved detail^35^ but even the evaluation of histological slices several hundred of micrometres thick cannot facilitate the quantitation of all of the vascular branching and connectivity existing within a sample. A superior approach would involve three-dimensional imaging to allow the visualisation, reconstruction and quantification of bone vasculature. However, µCT lacks the resolution and sensitivity required to achieve such an objective. Blood vessels have been previously visualised in cortical bone using high-resolution synchrotron-based computed tomography at the TOMCAT beamline of the Swiss Light Source^36^; however access to any synchrotron is often time limited and financially prohibitive in many cases. In this study, we have quantified the effects of different VEGF delivery methods, across treatment groups, using a ‘synchrotron like’ lab-based system that has enabled a novel use for X-Ray microscopy and quantification of the intraosseous vascular space to visualise and quantify bone vascular branching. The approach demonstrated that VEGF transfection *in vivo* could increase intraosseous branching that was quantifiable *ex vivo*. It is possible that porous structures found within calvariae by XCT could include spongey diploe; however, this would be unusual as the branching patterns observed are highly characteristic of vasculature. Future experiments could include the use of nanoparticle-based or heavy-metal-based contrast agents to highlight the soft tissue of the vasculature within calvariae. Importantly, this technique offers wide potential in applications such as quantification of blood vessels in other bone types in addition to calvariae or, angiogenesis, within calcified tumours such as osteosarcoma.

In conclusion, direct delivery of VEGF to bone promises a useful approach for the enhancement of angiogenesis and osteogenesis. However, the precise effect depends significantly on the delivery mechanism employed. A transient single dose of recombinant protein enhances expansion of bone formation while a steady release of VEGF, following transfection, promotes angiogenesis. Confirmation of the mechanisms at play and the optimal delivery approach auger well for improved bone health for an ageing population.

## Methods

### Cell culture and transfection

For the preparation of RNA used in transfection, the full cDNA of human VEGF165A and EGFP was reverse translated and codon optimised for maximal human expression. This was *de novo* synthesised (Eurofins MWG) and cloned into a standard vector. mRNA DNA templates were generated by PCR. HiScribe T7 ARCA mRNA kits (NEB) were used to generate modified mRNAs containing 5mCTP and Pseudo-UTP. These also contained an anti-reverse cap anolg (ARCA) and a polyA tail according to the kit. The modified nucleotides have been shown to suppress RNA-mediated innate immune activation *in vivo*. DNase I and LiCl were employed for DNA template removal and rapid mRNA purification. Human osteoblast-like MG-63 osteosarcoma were from ATCC and all cell culture reagents supplied by Thermo Fisher Scientific unless specified. MG-63 cultures were maintained in T75 flasks containing complete media of 10% FBS in DMEM with 100 U/mL penicillin and 100 µg/mL streptomycin (under standard cell culture conditions at 37 °C, 5% CO_2_). Cells were passaged when cultures reached 70% confluence. For transfection, 40,000 cells were plated per well in complete media in a 24 well plate. After 24 hours, the complete media was removed and cultures were rinsed twice with 1x PBS before being replaced with 250 µl of OptiMEM per well. For each well a transfection solution was prepared in two stages. A solution of 1 µg either *VEGF*_165_ or *GFP* RNA and 25 µl OptiMEM was rested at room temperature for 5 minutes while a solution of 0.75 µl of lipofectamine 2000 and 25 µl OptiMEM was prepared. Both solutions were combined and incubated at room temperature for 20 minutes to allow lipofectamine-RNA complexes to form and 50 µl of transfection solution was added to each well of a 24 well plate. Cells were incubated in transfection solution in OptiMEM for 5 hours. OptiMEM was used without FBS or antibiotics to enhance transfection efficiency and minimise cell death, which can otherwise be exacerbated in combination with lipofectamine. Cultures of transfected MG-63_*GFP*_ and MG-63_*VEGF*165_ cells were rinsed with 1x PBS and returned to complete medium and GFP protein expression was checked after 16–20 hours with a Zeiss Axiovert 200 M inverted microscope as positive control for successful transfection at which point plates were then divided according to experiment.

MG-63 cells were seeded onto electrospun PCL scaffolds generously provided by J.Puetzer and M. Stevens (Imperial, UK). PCL discs cut using a 5 mm biopsy punch were sterilised in 70% ethanol and then under UV light. PCL discs were incubated with 20% BSA in 1x PBS for 1 hour to reduce hydrophobicity and enhance cell adhesion prior to cell seeding. To prevent floating, PCL discs were held down by insect pins positioned into a pre-prepared polydimethylsiloxane lining at the bottom of each well. MG-63 cells were plated at a density of 200,000 per PCL disc in 100ul complete media for each and incubated for one hour to allow cell attachment before topping up wells with media. For transfection, reagents were scaled up in proportion with cell number so that 5ug of RNA and 3.75ul lipofectamine was used per PCL disc.

### Cell proliferation

The amount of DNA present was used as an indicator of differences in cell proliferation between treatment groups. Cells were lysed and freeze thawed 24 hours after transfection to release DNA which was quantified with the Quant-iT PicoGreen dsDNA Assay Kit (Thermo Fisher Scientific) according to the manufacturer’s instructions.

### RNA extractions, cDNA synthesis and quantitative PCR

RNA was extracted 24 hours after transfection with the RNeasy plus minikit (Qiagen) following manufacturer’s instructions. RNA quantity and quality were evaluated with a Nanodrop Spectrophotometer (Labtech). cDNA synthesis was carried out with Superscript Vilo kit (Thermo Fisher Scientific). Gene expression was quantified by quantitative PCR. Each 25 µL reaction contained 12.5 µl SYBR Green PCR Master Mix (Thermo Fisher Scientific), 250 nM of each primer, and 1ul cDNA. All reactions were carried out in triplicate on an AB7500 Real-Time PCR system (Applied Biosystems). Initial activation was at 95 °C for 10 minutes, followed by 40 cycles of 95 °C for 15 seconds and 60 °C for 60 seconds. The 2-ΔΔCt method was used for the relative quantification of gene expression in MG-63_*VEGF*165_ cells compared to MG-63_*GFP*_ cells, and data were normalized to β-actin expression. Primer sequences used were VEGF forward CACACAGGATGGCTTGAAGA, VEGF reverse AGGGCAGAATCATCACGAAG, β-actin forward GGCATCCTCACCCTGAAGTA and β-actin reverse AGGTGTGGTGCCAGATTTTC.

### VEGF ELISA

MG-63_*GFP*_ and MG-63_*VEGF*165_ cells in 24 well plates or on PCL scaffolds were rinsed three times with 1x PBS and incubated in serum free medium for 24 hours. Conditioned media samples were collected and quantified using the Human VEGF Quantikine ELISA Kit (RnD Systems, UK) according to the manufacturer’s instructions.

### CAM assays

All egg chorioallantoic membrane (CAM) studies were undertaken following approval from the local Animal Welfare and Ethics Review Board (AWERB) University of Southampton and carried out in accordance with the guidelines and regulations stipulated in the Animals (Scientific Procedures) Act, UK 1986 under the approved Home Office Project license (PPL 30/2762). CAM assays were performed based on previously established protocols^37,38^. Briefly, procedures began on eggs 10 days after fertilisation. Windows were made into the eggshell through which the CAM could be visualised. PCL scaffolds with either MG-63_*GFP*_ cells, MG-63_*VEGF*165_ cells or 10 µL of rhVEGF_165_ (1 µg/mL) were placed directly on to the CAM, windows were sealed with sterile parafilm and eggs incubated without rotation for a further 7 days. On day 17 CAM membranes were harvested and fixed in 4% PFA in 1x PBS. Angiogenesis in the CAM was quantified using a dissecting microscope with an eyepiece Chalkley graticule, which has been previously used to provide measurements of vascular density in the CAM assay^39^.

### VEGF delivery *in vivo*

All mouse *in vivo* studies were undertaken following approval from the local Animal Welfare and Ethics Review Board (AWERB) University of Southampton and carried out in accordance with the guidelines and regulations stipulated in the Animals (Scientific Procedures) Act, UK 1986 under the approved Home Office Project license (PPL 30/2880).

A transfection solution containing 5 µg of *GFP* or *VEGF*_*165*_ RNA and 3.75 µl lipofectamine MessengerMAX made up to 10 µL total volume in 1x PBS was prepared 20 minutes prior to each procedure to provide an optimal time for RNA-lipofectamine complexes to form. Control solutions were made with rhVEGF_165_ (1 µg/mL) in 1x PBS. Age-matched wild type MF-1 mice were anaesthetised with fentanyl-fluanisone (Hypnorm; Janssen-Cilag Ltd.) and midazolam (Hypnovel; Roche Ltd.) in sterile water at a ratio of 1:1 and a dose of 10mL kg-1 intraperitoneally. A lengthways incision was made along the skull so that skin could be pulled back to expose the calvariae and any remaining soft tissues were scraped away. Solutions containing either RNA-lipofectamine complexes or rhVEGF_165_ protein were directly pipetted onto the exposed calvariae prior to suturing. Mice were incubated at 37 °C on heat mats for recovery before being returned to cages provided with mouse chow and water *ad libitum*.

### *In vivo* µCT

Scans were performed on a Skyscan 1176 *in-vivo* CT scanner (Bruker) at 65 kV, 385 µA with a 1 mm thick Aluminium filter. Exposure time was 135 ms with a 0.5°rotation step to achieve an 18 µm voxel size. Tomograms were reconstructed using NRecon (version 1.7.1.6, Bruker). The scan at the time of surgery was set as reference and each subsequent acquisition was 3D registered for perfect alignment to the reference using Dataviewer (version 1.5.1.2, Bruker). Datasets were analysed in CTAn (version 1.16.4.1, Bruker). The defined ROI was used in subsequent scans to determine bone development at the calvarial site. As greyscale of 200 or more corresponds to bone over 1 g/cm^3^ bone mineral density, high density bone volume was calculated using greyscale values 200–255.

### X-ray micro computed tomography and analysis

Mice were culled on day 42 and isolated calvariae subsequently fixed in 4% paraformaldehyde for 48 hours and transferred to 70% ethanol prior to scanning. Isolated calvariae were imaged using high-resolution X-ray computed tomography (XCT) (ZEISS Xradia 520 Versa, Carl Zeiss X-ray Microscopy, Pleasanton, CA, USA) set to operate at 60 kV, 5 W and an LE1 filter positioned directly after the X-ray source. X-ray projection images (2001 images) were collected over 360°at equal intervals with an isotropic voxel size of 3.15 µm (exposure time per projection was 6 seconds). Projections were then reconstructed using the manufacturer’s integrated software (Scout and Scan Reconstructor, Carl Zeiss X-ray Microscopy, Pleasanton, CA, USA), which utilises a filtered back projection reconstruction algorithm. The reconstructed tomograms, 16-bit grey-level images, were analysed double blinded using Avizo 9.3 (Thermo Fisher Scientific) for image segmentation and quantification. Calvarial vasculature was segmented from surrounding bone by isolating the bone into a binary image using the Interactive Thresholding tool. The grey-level range for thresholding was manually determined for each sample and selected to ensure that the entire bone was included within the initial segmentation. The binary image of the bone was applied back to the full image volume and used to mask the bone area for an Interactive Thresholding to segment the bone vasculature. The binary image of the vasculature was used for quantitative analysis by implementing the Volume3d measurement tool. Only the vasculature that was fully contained within the bone was considered for analysis. The vasculature network and connectivity were constructed using the Auto-Skeleton tool contained within the software. The number of branching nodes (3 of more branches) was normalised to bone volume within the ROI.

### Statistics

Statistics were carried out using GraphPad Prism 8 with additional analysis in IBM SPSS Statistics 25. Normality tests were carried out in GraphPad Prism 8. In analysis comprising two groups for comparison, significance was determined using a t-test. Where more than two groups were compared, significance was determined using ANOVA with appropriate post-hoc tests. Univariate Analysis of Variance and differences between individual treatment groups determined using Tukey post-hoc tests in IBM SPSS Statistics 25. Figures were generated in GraphPad Prism 8 and data presented with individual data points and mean with standard deviation error bars.
